# Supplementary material for: Identifying and Prioritising Behaviours to Slow Antimicrobial Resistance
Source: Antibiotics (Basel). 2023 May 23;12(6):949. doi: 10.3390/antibiotics12060949 (PMC10294905; doi:10.3390/antibiotics12060949)
Supplement: Supplementary file 1 [file antibiotics-12-00949-s001.zip › antibiotics-2397562-supplementary.pdf]

Supplementary Table 1: Behaviour descriptions given to broader community participants. For full survey please contact authors.

Community Behaviour Classification	Summary	Additional description included for survey participants
human use of antibiotics	Listen to your doctor. Do not “pressure” your doctor for antibiotics.	Patients asking their doctor for antibiotics may influence medical decision making (i.e., prescribing antibiotics when they are not needed), and contribute to AMR.
	Only take antibiotics when they are prescribed to you by a doctor	Using leftover antibiotics (either someone else’s, or from a previous prescription) may contribute to AMR.
	Returning unused antibiotics to a pharmacy.	Discarding leftover antibiotics helps remove them from the house (and potential further use). However, discarding them via the general waste stream may result in them entering the environment. The general community can take unused antibiotics to a pharmacy (which can dispose of them safely).
antimicrobial marketing on cleaning products	When shopping for personal cleaning products (e.g., hand soaps, body wash) choose products that do not promote antimicrobial agents.	These brands are less likely to contain antimicrobial agents that contribute to AMR.
	When shopping for household cleaning products (e.g., multi-purpose cleaner, dishwashing liquid) choose products that do not promote antimicrobial agents.	These brands are less likely to contain antimicrobial agents that contribute to AMR.
	Encourage the people close to you (e.g., family and friends) to choose personal and household cleaning products that do not promote antimicrobial agents.	People’s purchasing behaviours are influenced by the opinions of people close to them. Also, when people encourage other people to adopt a behaviour, they themselves may be more likely to continue to engage in the behaviour.
	At your workplace, ask procurement (e.g., the person responsible for purchasing cleaning products at your workplace) to choose personal and household cleaning products that do not promote antimicrobial agents.	Workplaces are high uses of cleaning products and so offer a further opportunity to reduce the amount of antimicrobial agents entering the environment.
	Contact your local council to find out where you can safely dispose of personal	Discarding cleaning products containing antimicrobial agents removes them from the home (and potential further use). However, discarding them via the general

	or household cleaning products that do promote antimicrobial agents.	waste stream may contribute to AMR. Local councils often provide a service to safely dispose of the cleaning products, or at least know where residents can go to safely dispose of them.
lobbying for change	Lobby the regulator, or product manufacturer, against antimicrobial marketing.	Cleaning product companies use antimicrobial marketing as a strategy to increase sales, resulting in increased use of these products, which may increase AMR as their products contain antimicrobial agents.
	Lobby the regulator, or product manufacturer, to remove antimicrobial agents from their cleaning products.	Cleaning product companies can avoid using antimicrobial agents in their cleaning products.
	Lobby supermarkets to only sell "antibiotic free" meat products.	Agricultural practices (e.g., overuse of antibiotics with livestock, through feeding practices or otherwise) can contribute to AMR. Lobbying supermarkets may help prevent the impact of these practices reaching the consumer, and may result in changing agricultural practices.
stopping the spread of bacteria	Wash your hands regularly.	Although regular hand washing may not directly prevent the development of AMR, it does help stop the spread of resistant microbes.
